# Considering Loxapine Instead of Clozapine: A Case Series and Literature Review

**DOI:** 10.7759/cureus.12919

**Published:** 2021-01-26

**Authors:** Shristi Shrestha, Raafae S Agha, Zershana Khan, Kaushal Shah, Shailesh Jain

**Affiliations:** 1 Psychiatry, Texas Tech University Health Sciences Center, Midland, USA; 2 Psychiatry, Griffin Memorial Hospital, Norman, USA

**Keywords:** loxapine, clozapine, sialorrhea, antipsychotics, noncompliance, atypical

## Abstract

This case series highlights the importance of loxapine in psychiatric disorders when clozapine is unable to control agitation and psychotic symptoms due to medication noncompliance, intolerable side effects, or inadequate clinical outcomes. Loxapine is classified as a first-generation typical antipsychotic but displays atypical antipsychotic-like characteristics with structural similarity to clozapine. However, since the inception of second-generation antipsychotic medications, the role of loxapine in modern psychiatric practice has become limited. Loxapine has been underutilized due to concerns of side effects and lack of in-depth research about its safety, tolerability, and efficacy. This report revisits loxapine’s clinical utility through two well-studied long-term inpatient cases, where it played a crucial role in controlling psychotic symptoms and achieving better medication compliance by eliminating significant barriers, including monitoring regular differential complete blood counts. We aim to add to the current evidence and literature about the advantage of using loxapine for specific clinical scenarios in psychiatric practice.

## Introduction

According to the 2018 National Survey on Drug Use and Health report, about 47.6 million people above 18 years of age had any mental illness in the past year, corresponding to 19.1% of adults in the United States. Its estimate includes 11.4 million adults with severe mental illness [1]. Agitation is a common symptom in psychiatric patients and is frequently found in psychotic disorders, schizophrenia, schizoaffective disorder, and bipolar I and II disorders [2]. According to the National Institute of Mental Health, the prevalence of schizophrenia ranges from 0.33% to 0.75% worldwide and 0.25% to 0.64% in the United States [3]. Bipolar disorder is estimated to be prevalent in 2.9% and 4.4% of US adolescents and adults, respectively [4].

As mental illness is highly prevalent in the United States, it is essential to manage agitation that usually manifests in the acute onset of common psychiatric disorders [2]. Historically, agitation is managed pharmacologically with the help of mostly barbiturates, benzodiazepines, and antipsychotics. After introducing antipsychotics in psychiatric clinical practice, it has become the preferred mainstay for controlling agitation and psychotic symptoms due to their minimal side effect profile, fast action, and better anticipated results compared to other traditional means [2]. Typical antipsychotics were widely used initially to manage agitation and mood disorders, but their role has been diminished against atypical antipsychotics in long-term treatment management due to the risk of dire extrapyramidal symptoms consisting of dystonia, akathisia, dyskinesia, and parkinsonian symptoms [2]. Second-generation antipsychotics such as clozapine have shown superior outcomes compared to other antipsychotics in treating psychosis and mood disorders [5]. However, it poses barriers to medication noncompliance due to unfavorable side effects or ineffectiveness. Some common side effects include sialorrhea, seizure, constipation, sedation, weight gain, and the hassle of regular mandatory blood monitoring for life-threatening adverse events. These circumstances warrant psychiatrists to look for alternative drug treatment [5].

Loxapine, first approved in 1975 by the US Food and Drug Administration (FDA), is a typical first-generation antipsychotic [6]. It is a dibenzodiazepine antipsychotic that blocks postsynaptic mesolimbic dopamine receptors in the brain [6,7]. It also possesses serotonin 5-HT2-blocking activity. As its chemical structure is very similar to clozapine, it exhibits several atypical characteristics. Loxapine has a higher affinity towards dopamine D3 than D2 receptor, and it binds with the D4 receptor in higher affinity than other dopaminergic receptors, which are similar to clozapine [6]. Primarily, its metabolism includes demethylation to amoxapine [7]. It is used to treat agitation, bipolar mania, and psychosis, as in schizophrenia. Unlike clozapine, it is also available in the form of oral, intramuscular, and inhalation route of administration. The FDA has also approved the inhalation form of loxapine to treat agitation associated with schizophrenia and bipolar disorder in adults [6,7]. The inhalation form of loxapine is contraindicated to patients with coexisting asthma and chronic obstructive pulmonary disease due to black box warning, which indicates that patients are at risk of serious or life-threatening adverse effects [8-10].

## Case presentation

Case 1

A 35-year-old Hispanic male was admitted to a state psychiatric hospital for aggressive behavior. He was found to be incompetent to stand trial for assaulting a public servant and was started on court-ordered medications. At the time of admission, he experienced command-type auditory hallucinations, where he was hearing voices that were telling him that people don’t like him and he needs to defend himself often. His auditory hallucination was also instructing him to hit staff physically. Occasionally, he complained about visual hallucinations, where he saw shadows. He was diagnosed with schizoaffective disorder marred with a history of violence. He was intensely aggressive towards staff. He permanently disabled one nurse in such an outburst. He was noncompliant with oral clozapine and would often refuse to take it. As a court-ordered mandatory substitute, he was involuntary given intramuscular chlorpromazine, olanzapine, haloperidol, and lorazepam instead of oral doses of clozapine. It was then identified that sialorrhea, which is defined as excessive salivation or drooling, was the prime reason for his refusal of clozapine.

We initiated oral loxapine, on which the patient started showing remarkable positive medication compliance and control of symptoms, including agitation, mania, depression, and sialorrhea. Loxapine was added to oral haloperidol and clonazepam that was started initially. The extrapyramidal syndrome (EPS) was addressed by substituting haloperidol with chlorpromazine and adding benztropine. With better compliance for loxapine, there was a significant drop in the patient’s aggressive and violent behaviors. As his aggressive behavior was under control, we were able to titrate down the dosing of clonazepam to reduce his daytime sedation. The final psychiatric medication regimen included loxapine 75 mg oral (PO) twice a day (BID), clonazepam 1 mg PO thrice a day (TID), chlorpromazine 50 mg PO BID, benztropine 1 mg PO TID, and sodium valproate 500 mg PO every night at bedtime (QHS).

Case 2

A 45-year-old African American female was declared incompetent to stand trial for a trespassing charge. She had multiple legal charges in the past 30 years, ranging from burglary, robbery, and assault. Initially, she was anxious, agitated, and had disorganized thoughts with loose associations. She complained of auditory hallucinations and heard several voices and crying of people. Upon further examination, she was found to be paranoid and reported being “abused by television and radio.” She firmly believed that other people could put thoughts in her mind. Also, she believed that it was her 12th birth in the form of reincarnation and that she was currently seven months pregnant, despite knowing that her urine pregnancy test was negative. The patient was on sodium valproate, quetiapine, and trazodone. Clozapine was listed in her past medication list but was discontinued due to unspecified side effects and medication compliance issues. However, she showed her displeasure towards clozapine due to weight gain, excessive salivation, and sedative effect.

There was some improvement on risperidone 2 mg BID over the next two weeks. However, she continued to have disorganized thinking, paranoia, and agitation, even with risperidone titrated to 4 mg BID. Due to continued psychotic symptoms, we introduced loxapine 25 mg BID along with benztropine 2 mg PO BID to prevent the EPS. Anxiety was moderately controlled with diphenhydramine 25 mg PO BID on a pro re nata (PRN) basis. With the initiation of loxapine in her regime, we observed better control in her agitation, thought process, and paranoia. Loxapine was uptitrated to 25 mg TID to optimally control her agitation. Complete remission of psychotic symptoms was noted after initiating loxapine. During her inpatient stay, she gained 64 pounds, along with hyperlipidemia and elevated glycated hemoglobin, which led us to introduce metformin 1,000 mg extended-release once daily for antipsychotic-induced weight gain, after which she did not gain any additional weight.

## Discussion

We reported two chronic psychotic patients who were responsive to loxapine and either could not tolerate or were noncompliant with clozapine. Medication compliance with loxapine was significantly increased due to a lack of severe side effects such as sialorrhea, which also helped control psychotic symptoms and agitation without needing regular complete blood count (CBC) monitoring.

The side effect profile of loxapine is comparable to first-generation antipsychotics at higher doses of greater than 50 mg per day [10]. However, when used in lower doses, its effect is similar to that of a second-generation antipsychotic [7,10]. Although loxapine is identical in structure and comparable in efficacy to clozapine, it also has many significant differences, such as loxapine does not require frequent blood monitoring for agranulocytosis due to differences in oxidation by human neutrophils as seen in clozapine [6]. Loxapine is available in oral, intramuscular, and inhalation forms. For the treatment of schizophrenia, the recommended starting dose for oral administration is 10 mg BID at a maximum dose of 250 mg per day. Intramuscular dosing ranges from 12.5 to 50 mg every four to six hours. For acute treatment of agitation associated with schizophrenia and bipolar disorder, the dosing is 10 mg once daily by inhalation [11].

Loxapine has been found to be very effective in psychotic symptom management. It does not require mandatory regular monitoring of CBC for agranulocytosis [6]. As it is categorized as a typical antipsychotic, metabolic profile monitoring is not mandated as recommended for the atypical antipsychotics [10]. Due to the favorably less side effect profile of loxapine compared to clozapine, patients tend to be more compliant with this form of oral medication [11]. Side effects such as sialorrhea, constipation, hypotension, weight gain, and agranulocytosis are minimal and tolerable in loxapine. It also reduces the need for emergency PRN medications for agitation, psychosis, or anxiety [10,11]. Loxapine has similar cost benefits compared to clozapine. Compared to other antipsychotics, the cost is very similar or lesser, which always favors its compliance and affordability [11]. Missed loxapine doses are managed well compared to clozapine, as it eliminates the need for CBC laboratory reports and does not need to be tapered up from the baseline dose [6,7,10,11]. Inhalational form of loxapine can be used in the emergency room setting to manage acute agitation and psychosis, which is less invasive and lets patients feel less like they are not in control of their situation. Inhalational loxapine costs around 100 US dollars, which is very affordable for the institution as well [11].

In both cases, the patients reported being satisfied with the new treatment plan, as they experienced no dire side effects and psychotic symptoms were better controlled. In the first case, the patient stopped complaining of drooling or excessive salivation once he was on loxapine that he otherwise experienced with clozapine. Due to the adequate control of the patient’s aggressive behavior, psychiatrists lowered the dosage of clonazepam, which helped the patient attain improved alertness. The staff reported improved cooperation in the patient’s daily activities and oral medication intake. In the second case, the patient’s medication history reported unfavorable side effects, unavailability to follow-up, and medication noncompliance on clozapine once she was released from the inpatient care. With oral loxapine initiation, the patient needed less occasional PRN anxiolytic, and was observed to have less agitation and remission of psychotic symptoms.

## Conclusions

Loxapine can be an excellent alternative to clozapine. It is comparable to clozapine in efficacy, cost-effectiveness, and a better side effect profile with more administration routes. It has superiority for immediate use as it provides a convenient administration route through the inhalation route. As psychiatric agitation and uncontrolled psychotic symptoms are major challenges in a psychiatric setting, psychiatrists and healthcare workers would benefit from this unique option. Further well-designed comparative studies should be conducted on loxapine and other antipsychotics to identify and uncover its optimal use.
